# Array comparative genomic hybridization and flow cytometry analysis of spontaneous abortions and mors *in utero *samples

**DOI:** 10.1186/1471-2350-10-89

**Published:** 2009-09-14

**Authors:** Björn Menten, Katrien Swerts, Barbara Delle Chiaie, Sandra Janssens, Karen Buysse, Jan Philippé, Frank Speleman

**Affiliations:** 1 Center for Medical Genetics, Ghent University Hospital, 9000 Ghent, Belgium; 2 Department of Pediatric Hematology and Oncology, Ghent University Hospital, 9000 Ghent, Belgium; 3 Department of Clinical Biology, Immunology and Microbiology, Ghent University, 9000 Ghent, Belgium

## Abstract

**Background:**

It is estimated that 10-15% of all clinically recognised pregnancies result in a spontaneous abortion or miscarriage. Previous studies have indicated that in up to 50% of first trimester miscarriages, chromosomal abnormalities can be identified. For several decades chromosome analysis has been the golden standard to detect these genomic imbalances. A major drawback of this method is the requirement of short term cultures of fetal cells. In this study we evaluated the combined use of array CGH and flow cytometry (FCM), for detection of chromosomal abnormalities, as an alternative for karyotyping.

**Methods:**

In total 100 spontaneous abortions and mors *in utero *samples were investigated by karyotyping and array CGH in combination with FCM in order to compare the results for both methods.

**Results:**

Chromosome analysis revealed 17 abnormal karyotypes whereas array CGH in combination with FCM identified 26 aberrations due to the increased test success rate. Karyotyping was unsuccessful in 28% of cases as compared to only two out of hundred samples with inconclusive results for combined array CGH and FCM analysis.

**Conclusion:**

This study convincingly shows that array CGH analysis for detection of numerical and segmental imbalances in combination with flow cytometry for detection of ploidy status has a significant higher detection rate for chromosomal abnormalities as compared to karyotyping of miscarriages samples.

## Background

10 to 15% of all clinically recognised pregnancies end up in a spontaneous abortion. It is estimated that chromosomal abnormalities are the underlying cause in up to 50% of these miscarriages [1,2]. The vast majority of these chromosomal abnormalities are numerical aberrations (~86%), including trisomies, monosomies and polyploidies. Other abnormalities are structural aberrations (6%), with in a minority of cases, an inherited unbalanced chromosomal aberration following meiotic segregation in a parent having a balanced translocation. In approximately 8% of cases, fetal loss is due to chromosomal mosaicism in the embryo [3].

Most clinically recognised spontaneous abortions occur between 7 and 11 weeks of gestation. However, it has been assumed that most products of conception are lost even before the pregnancy is clinically recognised as such. These preclinical losses are due to failure of development, early arrest in cell division or implantation failure of the developing embryo.

Although the recurrence risk of numerical imbalances is very low in case of normal parental karyotypes, cytogenetic investigations of miscarriages may be invaluable as they may eliminate further testing and provide a better recurrence risk estimate for the couple. Classical cytogenetic investigations however, require viable tissue. As a result, in up to 40% of cases, no reliable karyotype can be obtained due to a high rate of culture failure. The occurrence of maternal contamination and the suboptimal quality of chromosome preparations are other limiting factors for chromosome analysis [4-6].

Other genetic techniques have been used to investigate products of conception, such as fluorescence in situ hybridization (FISH), multiplex-ligation dependent probe amplification (MLPA) and quantitative fluorescent PCR [2,7-9]. Although the majority of chromosomal aberrations can be detected with these techniques, structural aberrations are often missed. By using comparative genomic hybridization (CGH), numerical and unbalanced structural chromosomal aberrations can be identified without the need of mitotic active cells. However, this technique is very labour intensive and has a limited resolution [10-15]. Moreover polyploidies, cannot be detected with this comparative method. This last limitation can be circumvented by the use of flow cytometry (FCM) [5]. FCM is an easy, rapid, accurate and inexpensive technique for the measurement of cellular DNA content [16].

Comparative genomic hybridization on DNA-microarrays (array CGH), or molecular karyotyping, has proven to be very helpful in the detection of constitutional and acquired chromosomal aberrations [17-19]. Molecular karyotyping is fast, does not require tissue culture and allows screening of the entire genome for imbalances at high resolution.

In this study, we illustrate the advantages of array CGH combined with flow cytometry in comparison to conventional karyotyping.

## Methods

### Samples

Hundred miscarriages and mors *in utero *samples were investigated by conventional chromosome analysis, flow cytometry and molecular karyotyping using ~1 Mb BAC microarrays. All samples were sent to the Center for Medical Genetics, Ghent University Hospital, Belgium for chromosome analysis. Ethical approval for this study was obtained through the Ghent University Hospital ethics committee. All patients gave consent for molecular cytogenetic investigations on the miscarriage and mors-in-utero samples. First trimester miscarriage samples are generally collected by curettage. The analyses for second trimester miscarriage and mors *in utero *samples (obtained by expulsion of the fetus *in toto*) were performed on fetal skin, placental and/or umbilical cord material. The majority of samples were from women with advanced age or with a history of multiple miscarriages.

### Cytogenetic analysis

All tissue samples were grossly examined, removing maternal blood and decidua. Analysis of G-banded metaphase chromosomes was performed on cultured tissue samples using standard procedures. Karyotypes were described according to the guidelines of the ISCN 2005.

### Flow Cytometry

For DNA content analysis, the Coulter^® ^DNA Prep™ Reagents Kit (Beckman Coulter, Fullerton, CA, USA) was used according to the manufacturer's recommendations. In brief, the tissue was passed through a 70 μM Nylon Cell Strainer filter (Ref: 352350, BD Biosciences (Discovery Labware, Two Oak Park, Bedford, MA 01730 USA)) to prepare single cell suspensions. Uncultured cells (5 × 10^5^) were exposed to DNA Prep LPR for 30 sec, followed by incubation with DNA Prep Stain for 15 min at room temperature.

The fluorescence was analyzed on a Beckman Coulter Cytomics FC 500 5-colour flow cytometer equipped with a 488 nm argon ion laser. Dot plots of the peak area versus height and the peak ratio (height/area) versus area were used for doublet discrimination and exclusion. A minimum of 500 events (after exclusion of doublets) was collected.

DNA content analysis included determination of the mean channel fluorescence and the coefficient of variation (CV) of the G0/G1 peak. In addition, the DNA-index, a measure for the degree of DNA content abnormality, was calculated by dividing the G0/G1 peak channel of the sample by the G0/G1 peak channel of normal DNA-diploid reference cells obtained from peripheral blood of healthy volunteers.

### Array CGH analysis

DNA was isolated from tissue samples using the QIAamp DNA Mini Kit (Qiagen), according to the manufacturer's instructions. Array CGH analysis was performed on an in-house produced 1 Mb BAC-array as described by Menten et al. [17]. The scan images were processed with ArrayPro software (TECAN) and further analyzed with our in-house developed and freely available software tool arrayCGHbase [20]. Reporters with a signal to noise ratio < 3 were excluded from the analysis. Circular Binary Segmentation (CBS) was used to detect possible chromosomal breakpoints and calculate an individual chromosome based median.

## Results

### Conventional cytogenetic analysis

Hundred products of conception were investigated with conventional cytogenetic analysis using G-banding [see Additional file 1]. In twenty-eight cases karyotyping was not possible due to culture failure. Normal karyotypes were found in 11 (male) and 44 (female) cases. In 17 cases an abnormal karyotype was detected. Chromosomal abnormalities included predominantly autosomal trisomies (n = 9) followed by sex-chromosome aberrations (n = 2; 45, X) and polyploidy (n = 3; triploid samples). One apparently balanced and one unbalanced aberration were identified. The male to female ratio for the samples with a normal chromosomal result was 0.25, which is suggestive for maternal contamination due to culture artefacts in approximately 33% of the investigated samples.

### Flow Cytometry

Flow cytometry, utilizing propidium iodide as intercalating dye, was used to quantify the cellular DNA content of single cells. Hundred samples were analyzed using this relatively simple and rapid technique. The results of 3 samples could not be interpreted due to the presence of a high percentage of apoptotic cells. Ninety-four samples showed a diploid DNA content whereas 3 samples were DNA-triploid (DNA-index = 1.45). In the latter 3 samples, the triploidy was confirmed by karyotyping.

### Array CGH

Array CGH was performed on all samples. In 23 cases an abnormal result was found. Chromosomal abnormalities included autosomal trisomies (n = 15), monosomy for the X-chromosome (n = 5) and three structural aberrations (a combined deletion and duplication probably resulting from an unbalanced translocation and two terminal deletions) (Table 1). Ten of these aberrations were not detected with conventional karyotyping. One balanced translocation could not be detected by array CGH but was however inherited from a normal mother and is therefore probably not causal for the spontaneous abortion.

**Table 1:** array CGH and FCM findings in 100 spontaneaous abortions/mors *in utero *samples

***finding***	***No of cases***
normal male profile	34
normal female profile	38
abnormal profile	26
trisomy 6	2
trisomy 13	1
trisomy 15	2
trisomy 16	2
trisomy 18	4
trisomy 21	4
monosomy X	5
triploidy	3
dup(13)(q32.1qter)del(20)(pterp12.1), male	1
del(7)(q36qter), female	1
del(X)(q28qter), female	1
failure	2

In two additional cases, a triploidy was suspected due to aberrant ratios for the sex-chromosomes. Due to poor DNA quality, no result could be obtained for 2 samples. In 34 cases a normal male result was obtained. Thirty-nine samples were of normal female origin giving a male to female ratio of 0.87.

In Table 2, results of array CGH and FCM are summarized according to the gestational age (per trimester). From this table it is clear that highest proportion of chromosomal aberrations can be found in 1st trimester miscarriages.

**Table 2:** array CGH and FCM findings per trimester

***trimester***^***§***^	***I*** ***(n = 50)***	***II*** ***(n = 34)***	***III*** ***(n = 16)***	***Total*** ***(n = 100)***
normal male	15	17	2	34
normal female	18	11	9	38
abnormal	17	6	3	26
failed	0	0	2	2
aberrant	~34%	~18%		~26%

§: I, II, III: respectively first, second and third trimester

In summary, 27 chromosomal abnormalities were identified in 100 investigated samples by using conventional karyotyping and array CGH in combination with FCM. This percentage is rather low compared to previous array CGH and conventional cytogenetic studies reporting chromosomal aberrations in up to 50% of spontaneous abortions [3,21-24]. The proportion of numerical versus structural chromosomal aberrations is comparable to previous array CGH based [22,24] and cytogenetic investigations [3]. Monosomy X was the most common observed aneuploidy (5) and trisomy 18 and 21 were observed as the most common trisomies. Other trisomies observed include chromosome 6, 13, 15 and 16 (Table 1).

## Discussion

In order to assess the feasibility of array CGH as an alternative to routine karyotyping we compared results obtained by both methods on a series of 100 samples from spontaneous abortions. Given the inability of array CGH to detect triploidy or tetraploidy we decided to complement analyses with flow cytometric assessment of DNA index. Our results show that array CGH/FCM clearly outperforms on the standard karyotyping assay. Array CGH/FCM was successful in almost all samples whereas karyotype failure was noted in as much as 28% of cases. In ten cases, array CGH was able to reveal an aberration whereas the karyotype was normal or failed.

Flow cytometry was able to identify all three triploid samples identified by conventional karyotyping. Array CGH showed aberrant ratios for the sex chromosomes for 2 out of 3 cases. However, the samples could not unambiguously be assigned triploid. Hence, a combined approach of array CGH and FCM or alternatively quantitative fluorescence PCR, is needed to unambiguously detect polyploidy.

Although this study thus clearly shows the advantages of array CGH versus karyotyping, other rapid molecular methods for detection of DNA copy number abnormalities such as fluorescence in situ hybridization (FISH), multiplex-ligation dependent probe amplification (MLPA) and quantitative fluorescent PCR could also be of value for analysing fetal wastage material [2,7-9]. Although the most frequent occurring chromosomal aberrations (e.g. trisomy 16, 21, 22) can be detected with these techniques, certain structural aberrations such as interstitial deletions can however still be missed. Although no interstitial deletions or duplications were detected in this study, it can be anticipated that larger follow-up studies (on higher resolution arrays) might unravel interstitial aberrations responsible for fetal loss.

One major obstacle in the analysis of abortion material is avoiding cultural artefact. The present study clearly shows that karyotyping suffers more from maternal contamination. We calculated that in ~33% of samples a normal female karyotype was found due to the presence of maternal tissue.

This percentage is comparable with other reports demonstrating that at least 30% of 46, XX results are due to contamination by maternal deciduas in first trimester abortion specimens [4].

With the advent of recent technical innovations, the increased availability of commercial microarray platforms and the ability to automate, prices of microarray analysis are decreasing drastically leading to a price-competitive platform compared to tissue culture and conventional karyotyping or other (molecular) cytogenetic techniques such as MLPA or QF-PCR. The introduction of high resolution oligonucleotide or SNP platforms may eventually lead to a higher pick-up rate of submicroscopic aberrations responsible for fetal loss.

## Conclusion

In conclusion, array CGH analysis for copy number variations together with FCM for ploidy status determination, enables a refined and more complete (molecular) cytogenetic analysis of spontaneous abortions. Because array CGH is not dependent on cell culture, reporting times can be significantly reduced. Furthermore, array CGH/FCM is less labour intensive and amenable to further automation, increasing throughput and reducing hands-on time. Therefore it is likely to become the method of choice in most clinical diagnostic laboratories, especially since array CGH has an increased resolution so that smaller unbalanced chromosomal aberrations can more easily be detected.

## Competing interests

The authors declare that they have no competing interests.

## Authors' contributions

BM conceived of the study, and participated in the design and coordination of the study and drafted the manuscript. KB carried out the array CGH studies. KS carried out the flow cytometry. BCD and SJ collected all necessary clinical information. JP and FS participated in the design of the study and helped to draft the manuscript. All authors read and approved the final manuscript.

## Pre-publication history

The pre-publication history for this paper can be accessed here:



## Supplementary Material

Additional file 1 Results of chromosome analysis, flow cytometry (FCM) and array CGH for all 100 samples (trim. = gestational age, I, II, III: respectively first, second and third trimester). Click here for file
