# Supplementary material for: Array comparative genomic hybridization and flow cytometry analysis of spontaneous abortions and mors in utero samples
Source: BMC Med Genet. 2009 Sep 14;10:89. doi: 10.1186/1471-2350-10-89 (PMC2753309; doi:10.1186/1471-2350-10-89)
Supplement: Additional file 1 — Results of chromosome analysis, flow cytometry (FCM) and array CGH for all 100 samples (trim. = gestational age, I, II, III: respectively first, second and third trimester). [file 1471-2350-10-89-S1.doc]

**Additional Table: Results of chromosome analysis, flow cytometry (FCM) and array CGH for all 100 samples (trim. = gestational age, I, II, III: respectively first, second and third trimester)**

| **No.** | **trim.** | **karyotype** | **FCM** | **array CGH** |
| --- | --- | --- | --- | --- |
| 1 | I | 46,XX | diploid | normal female |
| 2 | I | 46,XX | diploid | dup(13)(q32.1qter)del(20)(pterp12.1), male |
| 3 | I | 46,XX | diploid | normal female |
| 4 | I | 46,XX | diploid | normal female |
| 5 | I | 46,XX | diploid | normal female |
| 6 | II | 46,XX | diploid | normal female |
| 7 | II | 46,XY,t(1;11) | diploid | normal male |
| 8 | II | 46,XX | diploid | normal female |
| 9 | I | 46,XX | diploid | normal male |
| 10 | I | no result | diploid | trisomy 6, female |
| 11 | III | 46,XY,+21 | diploid | trisomy 21, male |
| 12 | II | no result | diploid | normal female |
| 13 | II | 46,XX | diploid | normal female |
| 14 | I | 46,XX | diploid | normal female |
| 15 | II | 46,XY | diploid | normal male |
| 16 | I | 47,XY,+16 | diploid | trisomy 16, male |
| 17 | II | 47,XX,+18 | diploid | trisomy 18, female |
| 18 | III | 46,XX | diploid | normal female |
| 19 | I | 47,XX,+13 | diploid | trisomy 13, female |
| 20 | I | 46,XX | diploid | normal female |
| 21 | I | 46,XX | diploid | normal female |
| 22 | I | 47,XY,+16 | diploid | trisomy 16, male |
| 23 | II | 45,X | diploid | monosomy X |
| 24 | I | 46,XX | diploid | normal female |
| 25 | I | 46,XX | diploid | trisomy 15, male |
| 26 | I | 46,XX | diploid | monosomy X |
| 27 | II | no result | diploid | normal male |
| 28 | I | 46,XX | diploid | normal female |
| 29 | I | no result | diploid | normal male |
| 30 | I | 47,XX,+18 | diploid | trisomy 18, female |
| 31 | I | 46,XX | diploid | normal female |
| 32 | II | 46,XX | diploid | normal female |
| 33 | II | 47,XX,+21 | diploid | trisomy 21, female |
| 34 | II | 46,XX | diploid | normal female |
| 35 | III | no result | diploid | normal female |
| 36 | II | no result | diploid | normal male |
| 37 | II | 46,XX | diploid | trisomy 21, female |
| 38 | III | no result | diploid | normal female |
| 39 | I | 46,XX | diploid | normal female |
| 40 | I | 46,XX | diploid | normal male |
| 41 | I | 46,XX | diploid | normal male |
| 42 | II | 46,XY | diploid | normal male |
| 43 | III | no result | NA | no result |
| 44 | III | no result | NA | no result |
| 45 | I | 46,XX | diploid | normal male |
| 46 | I | 46,XX | diploid | normal male |
| 47 | I | 46,XX | diploid | normal male |
| 48 | I | 46,XX | diploid | normal male |
| 49 | I | 69,XXY | triploid | triploid? |
| 50 | III | no result | diploid | trisomy 21, male |
| 51 | II | 46,XY | diploid | normal male |
| 52 | III | no result | diploid | normal female |
| 53 | II | no result | diploid | normal male |
| 54 | II | 46,X,del(X)(q?qter)dn | diploid | del(X)(q28qter)dn, female |
| 55 | I | 46,XX | diploid | normal male |
| 56 | II | 46,XY | diploid | normal male |
| 57 | I | 46,XY | diploid | normal male |
| 58 | II | no result | diploid | normal male |
| 59 | II | no result | diploid | normal male |
| 60 | III | 47,XY,+18 | diploid | trisomy 18, male |
| 61 | II | 46,XX | diploid | normal male |
| 62 | II | 46,XY | diploid | normal male |
| 63 | II | no result | NA | normal male |
| 64 | I | 47,XY,+6 | diploid | trisomy 6, male |
| 65 | I | 46,XY | diploid | normal male |
| 66 | II | 46,XX | diploid | normal male |
| 67 | I | 69,XXX | triploid | normal female |
| 68 | II | no result | diploid | normal female |
| 69 | I | 46,XX | diploid | normal male |
| 70 | I | 46,XX | diploid | normal female |
| 71 | I | 46,XX | diploid | normal female |
| 72 | III | no result | diploid | normal male |
| 73 | III | no result | diploid | normal female |
| 74 | II | no result | diploid | normal male |
| 75 | I | 46,XX | diploid | normal female |
| 76 | II | no result | diploid | normal male |
| 77 | I | 46,XX | diploid | normal male |
| 78 | III | no result | diploid | normal male |
| 79 | II | 46,XX | diploid | normal female |
| 80 | I | 46,XX | diploid | monosomy X |
| 81 | I | no result | diploid | monosomy X |
| 82 | I | 46,XY | diploid | normal male |
| 83 | I | 46,XX | diploid | del(7)(q36qter), male |
| 84 | II | 46,XX | diploid | normal female |
| 85 | III | no result | diploid | normal female |
| 86 | II | no result | diploid | trisomy 18, male |
| 87 | I | 46,XX | diploid | normal female |
| 88 | II | 46,XX | diploid | normal female |
| 89 | III | no result | diploid | normal female |
| 90 | III | no result | diploid | normal female |
| 91 | I | 45,X | diploid | monosomy X |
| 92 | I | 46,XY | diploid | normal male |
| 93 | I | no result | diploid | normal female |
| 94 | I | 47,XX,+15 | diploid | trisomy 15, female |
| 95 | I | no result | diploid | normal female |
| 96 | I | 46,XX | diploid | normal female |
| 97 | II | 46,XY | diploid | normal male |
| 98 | II | 46,XY | diploid | normal female |
| 99 | III | 46,XX | diploid | normal female |
| 100 | I | 69,XXY | triploid | triploid? |
